# Imitative inhibitory control is associated with psychotic experiences in a sample from the general population

**DOI:** 10.3389/fpsyt.2024.1470030

**Published:** 2024-11-11

**Authors:** José Luis Ulloa, Daniel E. Núñez, Pablo A. Gaspar, Marcel Brass

**Affiliations:** ^1^ Programa de Investigación Asociativa (PIA) en Ciencias Cognitivas, Centro de Investigación en Ciencias Cognitivas (CICC), Facultad de Psicología, Universidad de Talca, Talca, Chile; ^2^ Millennium Nucleus to Improve the Mental Health of Adolescents and Youths (Imhay), Santiago, Chile; ^3^ Departamento de Psiquiatría y Salud Mental, Facultad de Medicina, Universidad de Chile, Santiago, Chile; ^4^ Departamento de Neurociencia, Facultad de Medicina, Universidad de Chile, Santiago, Chile; ^5^ Berlin School of Mind and Brain, Humboldt University of Berlin, Berlin, Baden-Württemberg, Germany; ^6^ Faculty of Psychology and Educational Sciences, Ghent University, Ghent, East Flanders, Belgium

**Keywords:** psychotic experiences, inhibitory control, imitation-inhibition, community sample, online study

## Abstract

Psychotic experiences (PE) are prevalent and associated with several negative mental health outcomes in both clinical and general population, particularly in young people. A promising avenue to understand the mechanisms underlying PE is to investigate functions that may be related to specific neural systems. One of these key cognitive mechanisms is the ability to control our imitative responses, which is strongly linked to an adequate social functioning. Emergent evidence suggests that impairments in this function might be involved in the early expressions of psychosis, but few studies have investigated its association with PE in a sample from the general population. Using an imitation-inhibition paradigm we examined this relationship in a community sample of young healthy individuals (N=204) and found that increased levels of PE levels were associated with lower imitative inhibitory control. These effects seem to be specific to imitation-inhibition as no correlation was found for a more general cognitive control as addressed by a Stroop-like task. In addition, these effects seem to be more evident for paranoid ideations. Overall, our results suggest that imitative inhibitory control can serve as a proxy to detect abnormalities associated with psychotic experiences.

## 1 Introduction

During the last years there has been an increased interest in developing tools to anticipate onset and predict prognosis in psychiatric disorders. In this line, researchers have started to look into stages previous to the clinical manifestation of a disorder. It has been proposed that psychotic symptoms exist on a continuum (1). On the most severe end are psychotic disorders, characterized by a set of symptoms that produce distress and impairment and that reach a clinically relevant threshold. On the least severe end there are psychotic experiences (PE), brief and attenuated manifestations of psychosis (paranoid ideas, bizarre thinking and perceptual abnormalities) that are observable in the general population (1). PE show a prevalence of 7% in general population (2) and appear particularly during childhood and adolescence (3). In most cases, PE are transitory and benign (4–6). Nevertheless, longitudinal studies have shown that persistent PE conveys an increased risk for later psychosis illness of about 3-16 times higher than the risk in adolescents with no PE (7, 8). Currently, PE are regarded as relevant risk factors for a wide range of psychiatric symptoms (9, 10) and negative behavioral and functional outcomes (11–14), particularly in young people (15). However, despite the increased interest in investigating these subthreshold phenomena (16), its nature and its role in the transition from mental distress to psychopathology are not well understood (17). One approach to gain a better understanding of the role of PE in the transition to psychopathology is to identify early signs associated with a shift toward a critical threshold (18) and to investigate behavioral, cognitive and neural aspects associated with changes in PE. There is evidence that motor dysfunctions are present in schizophrenia and may even emerge before a fully developed clinical state is reached (19–21). Impaired motor functions are observed in schizophrenia independent of medication (22) and are also present in healthy relatives (23). Failures in motor development can predict the onset of schizophrenia (24) and neuroimaging studies have revealed altered activity in motor-related brain regions associated with psychosis (25–28). Additionally, abnormalities in movement production and action processing, such as intentional binding and response monitoring, seem to indicate early dysfunctions before the full onset of psychosis (29–34). Taking all together, these studies suggest that motor impairments are potential predictors for transition to psychosis (35–37). Imitation is a distinctive sensorimotor process involved in the ability to control bodily movements. It has been suggested to play an important role in language acquisition, skill learning, socialization and enculturation (38). Beyond imitation, the capacity to inhibit imitative responses—imitative inhibitory control—seems to be essential for effective social interaction (39). Schizophrenia, a condition marked by significant alterations in social and emotional functioning, has been linked to deficits in these areas (40, 41). Taken together, these observations suggest that inhibitory processes related to person perception may be particularly impaired in schizophrenia, potentially serving as an early indicator of the disorder. There are incipient studies tackling the relationship between imitative inhibitory control and psychopathology. But these studies have found mixed evidence. One study found spare imitative inhibitory control but increased imitative behavior in patients with schizophrenia relative to healthy controls (42). In another study, impaired control of imitation was observed in patients with schizophrenia compared to healthy controls (43). However, little is known about imitative inhibitory control in people presenting subthreshold PE. The presence of PE in healthy populations offers unprecedented opportunities to investigate threshold signs of psychopathology without the interference of medication. Accordingly, we examined the associations between PE and imitative inhibitory control in a sample from the general population. We used a well-suited paradigm to study imitative inhibitory control: the imitation-inhibition paradigm (44, 45). In this task, participants have to respond to an imperative cue while an irrelevant finger movement occurs in the background - covertly affecting participants’ responses. Unlike other paradigms associated with the inhibition of responses (such a Go/No-go or Stroop) this task has an intrinsic social component (46, 47, for a review see 48). In the imitation-inhibition task, simple measures of reaction time and accuracy can be used to track changes in the control of imitation. This is in line with previous studies using reaction time measures in fast decision tasks to reveal relationships between schizophrenia and other complex processes, such as decision-making (49, 50). Given previous evidence linking disturbances of imitation and control of imitation in schizophrenia patients (43, 51) and under the general framework of the continuum of psychosis (1, 52) we expect to find a negative relationship between the imitative inhibitory control and PE levels. We will also examine the specificity of this association using a Stroop-like task. Given previous evidence of the role of inhibitory modulations in psychosis (37) we expect to find associations for this task as well.

## 2 Materials and methods

### 2.1 Participants

An initial sample of 211 participants was recruited. Participants were recruited online through advertisements in relevant social media groups on Instagram (instagram.com/psiquislab/). We recruited participants using a convenience sampling method, primarily leveraging the contacts of our research team and its network. Were eligible to participate in this study those who have consulted the Clinical Hospital at the University of Chile or that had contacted the PsiquisLab research team. Individuals who expressed interest in participating in the study were instructed to send an email to the designated study email address. Then, a research assistant reached out to these potential participants via email. During email exchanges, the research assistant explained the inclusion and exclusion criteria of the study. The inclusion criteria for the study were being between the ages of 13 and 30 years old. The exclusion criteria included a prior diagnosis of neurological disease or brain damage, a history of hard drug use (excluding alcohol), a diagnosis of suicidality, or a diagnosis of psychotic spectrum disorders. From the 211 participants we eliminated all those who consumed hard drugs. Those consuming light drugs (marihuana) were kept as long as its level of consumption was occasional (this is lower than once per month). After applying this criterion 204 volunteers remained in the final sample (155 women; mean = 22.3 years old, SD = 3.4; range = 14–30). All participants gave their informed consent and received compensation (CLP$ 4.000) for their involvement in the study. The study protocol and procedures were approved by the Ethics Review Board of the University of Talca (Code: 24-2019-E).

### 2.2 Demographic questionnaire

Participants were asked to complete a series of demographic questions, including age, gender (female or male), whether they belonged to an indigenous group and if so, which one, who they lived with (alone, parents, partner, other relatives, friends, or other), whether they had children (yes or no), whether they worked (yes or no), and their monthly income (less than CLP$99.000, between CLP$100.000 and CLP$299.000, between CLP$300.000 and CLP$499.000, between CLP$500.000 and CLP$799.000, between CLP$800.000 and CLP$1.000.000, or more than CLP$1.000.000). They were also asked about their education level (primary, secondary, technical/professional, incomplete university, complete university, or postgraduate), alcohol consumption (yes or no) and if so, how often (every day, weekends and sometimes during the week, only weekends, once a week, once a month, or less frequent), drug use (no, marijuana, hallucinogens, amphetamines, cocaine, crack, solvents, or other drugs) and if so, how often (every day, weekends and sometimes during the week, only weekends, once a week, once a month, or less frequent). Finally, they were asked if they had ever received psychological or psychiatric treatment (yes or no), if anyone in their family had received psychological or psychiatric treatment (never, currently, or in the past), if any close family member had attempted suicide (yes, no, or don’t know), and if any close family member had died by suicide (yes, no, or don’t know).

### 2.3 The Community Assessment of Psychic Experiences-Positive Scale

To assess PE we used the Community Assessment of Psychic Experiences-Positive Scale (CAPE-P15; 53), a worthwhile and validated tool for evaluating psychotic experiences (54). This is a 15-item self-report questionnaire addressing paranoid ideation (PI; 5 items), bizarre experiences (BE; 7 items), and perceptual anomalies (PA; 3 items). Responses to items range from 1 (never) to 4 (almost always). Higher CAPE scores reflect greater PE. CAPE total scores and subscale scores have shown good internal consistency in adolescents from Chile (McDonald’s ω = 0.91, PI = 0.77, BE = 0.83, and PA = 0.88; 55). In our sample, the internal consistency of the CAPE-15 total scores was good (α = 0.81; ω = 0.85), while the internal consistency was good for the PI (α = 0.76; ω = 0.81), good for the BE (α = 0.67; ω = 0.8) and acceptable for the PA (α = 0.61; ω = 0.66) subscale scores. The CAPE-P15 questionnaire was administered online hosted on the Redcap platform (https://www.project-redcap.org/).

### 2.4 Other questionnaires

We applied two more questionnaires to characterize our sample. We assessed psychological distress (three dimensions: depression, anxiety, and stress) using the Depression Anxiety Stress Scales (DASS; 56). The DASS 21 is a shortened version of the DASS that includes 21 items, with 7 items for each dimension. The DASS 21 is a self-report measure that asks individuals to rate the frequency and severity of various negative emotional and cognitive states over the past week. The DASS 21 has been found to be a reliable and valid measure of psychological distress. It has been validated for use with a variety of different populations, including adults, children, and older adults. The internal consistency of the Chilean version of the DASS-21 is reliable (α > 0.70 in all DASS’ subscales; 57). We also assessed nighttime and daytime components of insomnia using the Insomnia Severity Index (ISI). A brief self-report instrument that comprises seven items that evaluate the patient’s perception of insomnia symptoms, sleep dissatisfaction, interference with daytime functioning, noticeability of sleep problems to others, and degree of worry caused by the sleep problem (58). The ISI uses a 5-point Likert scale to rate each item (0 = no problem; 4 = very severe problem), yielding a total score ranging from 0 to 28 (58). The Spanish version of the ISI has demonstrated good psychometric properties and has been used in research studies with Chilean populations (59). The ISI has shown high discriminant validity in identifying insomnia (AUC = .97–.98) and an ISI score of ≥7 maximized sensitivity (.94–.97) and specificity (.88–.91) in detecting clinically significant insomnia. These questionnaires were administered online hosted on the Redcap platform (https://www.project-redcap.org/).

### 2.5 The imitation-inhibition task

To assess imitative inhibitory control, we used the online version of imitation-inhibition task (60). At the start of each trial, a message appeared instructing the participant to hold down the “G” and “H” keys on a keyboard using their right index and middle fingers. Once the participant had placed their fingers, the task began. Each trial started with a fixation cross, which appeared in the center of the screen for 1–1.5 s, followed by an image of a hand in a resting position for 1 s. This was followed by the simultaneous presentation of a number (either 1 or 2) between the two fingers and the lifting of one of the observed fingers (either the index or middle finger). Participants were instructed to lift their index finger for a 1 and their middle finger for a 2. The observed finger movements may either match (congruent trials) or mismatch (incongruent trials) the instructed finger movements. There was also a “neutral” condition in which a number was displayed but no finger movement occurred. The image remained on the screen until the participant responded or for 1.4 s, whichever occurred first. Finally, after a variable inter-trial interval (ITI) of 1–2 s, the next trial began. There were a total of 150 randomized trials, with 50 trials in each condition (congruent, incongruent and neutral). Before the main task, participants completed 10 familiarization trials. We recorded reaction times (RT) and number of errors.

### 2.6 The Stroop-like task

To assess general inhibitory control, we used an online task based on the Stroop task (61). In this task, participants are asked to imagine wearing colored rings in distinct fingers: a red (index finger) and a green (middle finger) one. They are asked to respond to colored imperative cues displayed on the screen. As before, at the beginning of a trial, participants are prompted to place their fingers on the keys of a keyboard and hold down the buttons. At this moment there is a reminder of the ring color-fingers mapping. This was followed by a fixation cross (1-1.5 sec), a still hand image and a colored imperative cue (appearing between the two fingers of the hand). When the stimulus displayed is a square, participants had to lift the finger that was matching the color of the imperative cue (congruent trials). When the stimulus is a rhombus, participants had to lift the finger that was not matching the color of the imperative cue (incongruent trials). After the subject’s response and a delay of 1.3 sec a blank screen is displayed. Finally, after a variable ITI of 1-2 sec the next trial began. There were a total of 120 randomized trials, with 60 trials in each condition (congruent and incongruent). Before the main task, participants completed 10 familiarization trials. We recorded reaction times (RT) and number of errors.

The experimental paradigms (imitation-inhibition and the Stroop-like tasks) were programmed using the jsPsych library (version 6.1.0) using a JavaScript framework for creating behavioral experiments (62). For the imitation-inhibition task we additionally used *ad-hoc* plugins (60). These experimental paradigms were hosted on the online platform Cognition.run (https://www.cognition.run/).

### 2.7 Procedure

Participants were instructed to complete the two experimental tasks described above and next a series of demographic questions and questionnaires (DASS, CAPE and ISI).

### 2.8 Data analysis

For data pre-processing we followed the procedures outlined by Westfal et al. (60). For RT analyses we excluded trials that were incorrect, that were below 100 ms and responses that were 3 standard deviations (SD) above the mean for each condition and participant. For error proportion (EP) analyses we converted the number of errors to proportions relative to the total of trials for each condition and for each participant. For the formal analysis we first evaluated the replicability of previous findings regarding the congruency effects in the imitation-inhibition and the Stroop-like tasks. We conducted two separate Wilcoxon tests (for the imitation-inhibition and the Stroop-like tasks) on RT and EP with congruency (congruent and incongruent) as a factor. Next, we investigated the relationship between PE and inhibitory control as reflected in each task. The key measure reflecting inhibitory control in each task is the congruency effect. The congruency effects were computed for each participant calculating the average RT or EP as a function of congruency (congruent and incongruent, separately for each task) and then computing the difference between these averages. The neutral condition in the imitation-inhibition task was included to create a comprehensive experiment but was not of interest when comparing effects between tasks. In the imitation-inhibition and the Stroop-like tasks a lower congruency effect reflects better inhibitory control. In the next step, we examined the relationship between PE and inhibitory control. We calculated total CAPE scores by averaging the subscale scores. Each CAPE subscale score was derived by averaging the responses to the relevant questions (sum of the item scores divided by the total number of items for that subscale). We then conducted Spearman correlation analyses between total CAPE scores and the congruency effects in both RT and EP for each task (imitation-inhibition and Stroop-like). Additionally, we explored specific associations between inhibitory control and individual CAPE subscales. For all analyses we evaluated the normality of the data using the Shapiro-Wilk test and selected appropriate parametric or non-parametric analyses accordingly. All analyses were performed using the R software.

## 3 Results

The sample consisted of 204 participants with an average age of 22.3 years (SD = 3.4), ranging from 14 to 30 years. The majority of participants were female (75.9%), with 155 females and 49 males. Most participants had incomplete university education (61.3%), followed by high school education (18.6%). A small number of participants identified as Mapuche (10) and as Aimara (1). Most participants lived with their parents (65.7%), while others lived with other family members (13.2%), alone (5.9%), with a partner (6.4%), with friends (2.9%), or in another living situation (4.9%). The vast majority of participants did not have children (96.6%). In this sample 61 participants (29.9%) were employed, and the most common monthly income categories were between CLP $300,000 and CLP $499,000 (23%) and between CLP $500,000 and CLP $799,000 (22.5%). The majority reported consuming alcohol (67%). Among those who consumed alcohol, the most common frequency was once a month (28.9%), followed by once a week (8.8%) and only on weekends (9.3%).

From the sample 66.7% of participants reported having received, at least once, psychological or psychiatric treatment in the past. Regarding family history of psychological treatment, 28.9% reported a family member currently receiving treatment, 25% reported that no family members had ever received treatment in the past, and 46.1% reported a family member has received treatment in the past. In the same line, 22.1% of participants reported a close relative attempting suicide in the past, while 53.4% reported no suicide attempts and 24.5% were unsure. In addition, in this sample 8.8% reported a close relative committing suicide, 88.2% reported no suicides, and 2.9% were unsure. Overall, about two-thirds of the sample had received psychological treatment, while rates of family history of treatment and suicide attempts/deaths were relatively lower.

**Table 1** presents the descriptive statistics for variables measured in the study, including the CAPE total scores and the specific CAPE subscales, including Paranoid Ideation, Bizarre Experiences, and Perceptual Anomalies. It also includes the total scores for the Depression Anxiety Stress Scales (DASS) and its subscales (Stress, Anxiety, Depression), as well as Insomnia scores from the Insomnia Severity Index.

**Table 1:** Descriptive statistics (Mean, Standard Deviation, and Range) for psychotic experiences (CAPE) and mental health measures (DASS, ISI).

	CAPE-T	CAPE-PI	CAPE-BE	CAPE-PA	DASS-T	DASS-S	DASS-A	DASS-D	ISI
Mean	1.3	1.7	1.3	1.1	42.9	15.4	13.3	14.2	13.0
SD	0.3	0.5	0.3	0.3	17.4	6.4	6.3	6.3	5.1
Range	1 - 2.6	1 - 4	1 - 2.7	1 - 3.3	21 - 96	7 - 33	7 - 32	7 - 35	5 - 28

CAPE-T, Total CAPE score; CAPE-PI, Paranoid ideation; CAPE-BE, Bizarre experiences; CAPE-PA, Perceptual anomalies; DASS-T, Total DASS score; DASS-S, Stress; DASS-A, Anxiety; DASS-D, Depression; ISI, Insomnia Severity Index.

### 3.1 Behavioral outcomes

**Figure 1:**
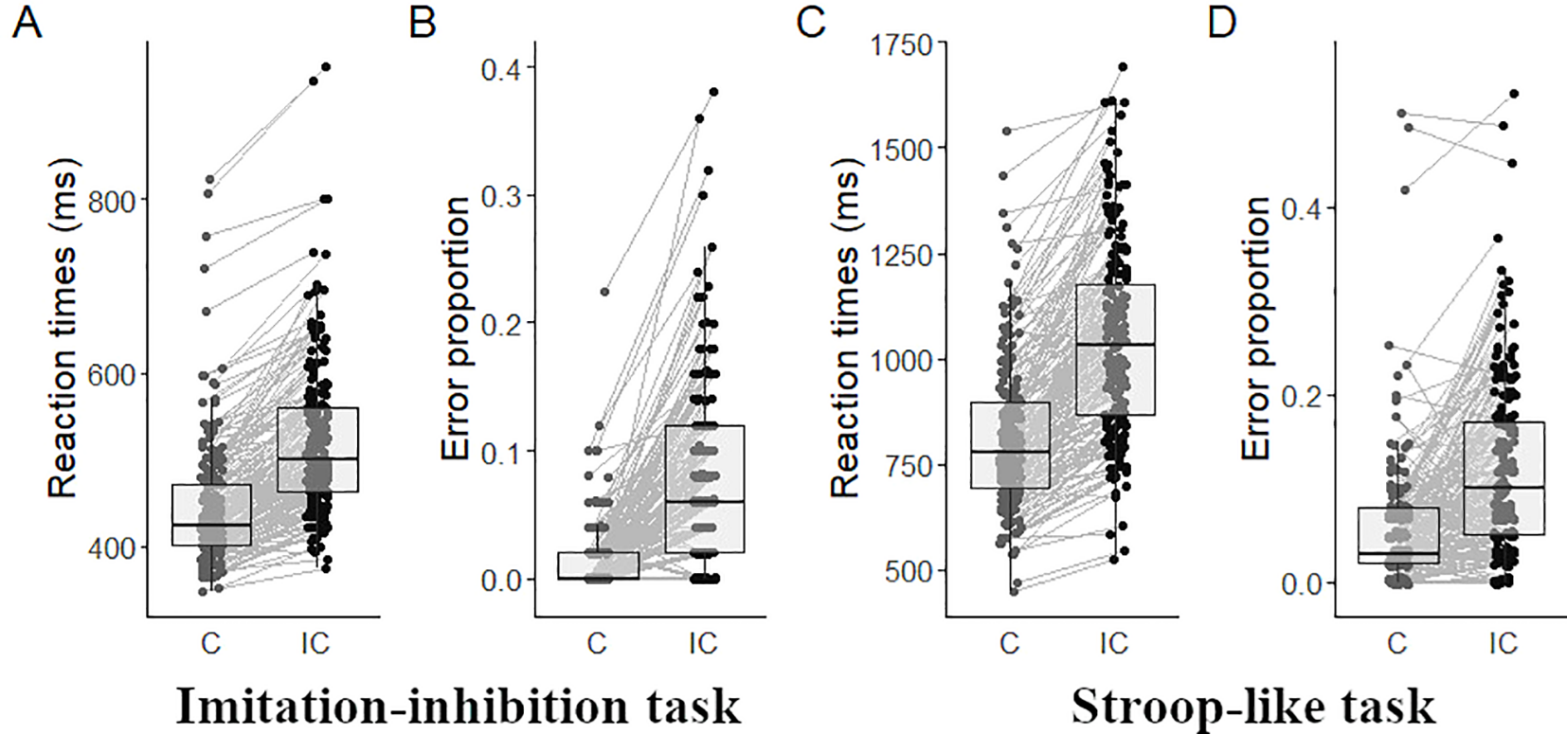
Behavioral outcomes. Mean RT in the imitation-inhibition **(A)** and the Stroop-like **(C)** tasks, and mean EP in the imitation-inhibition **(B)** and the Stroop-like **(D)** tasks as a function of experimental conditions. Each point represents a single participant. C, congruent; IC, incongruent.

In the imitation-inhibition task participants responded faster during congruent (444 ± 72; mean ± SD) than to incongruent trials (521 ± 87, W = 9, p < 0.001, **Figure 1A**) and showed fewer EP in congruent (0.01 ± 0.03) than in incongruent trials (0.08 ± 0.07, W = 193, p < 0.001; **Figure 1B**). In the Stroop-like task participants responded faster during congruent (811 ± 174) than to incongruent trials (1041 ± 228, W = 224, p < 0.001, **Figure 1C**), and showed fewer EP in congruent (0.06 ± 0.07) than in incongruent trials (0.12 ± 0.09, W = 1907, p < 0.001; **Figure 1D**).

### 3.2 Relationship between inhibitory control and psychotic experiences

**Figure 2:**
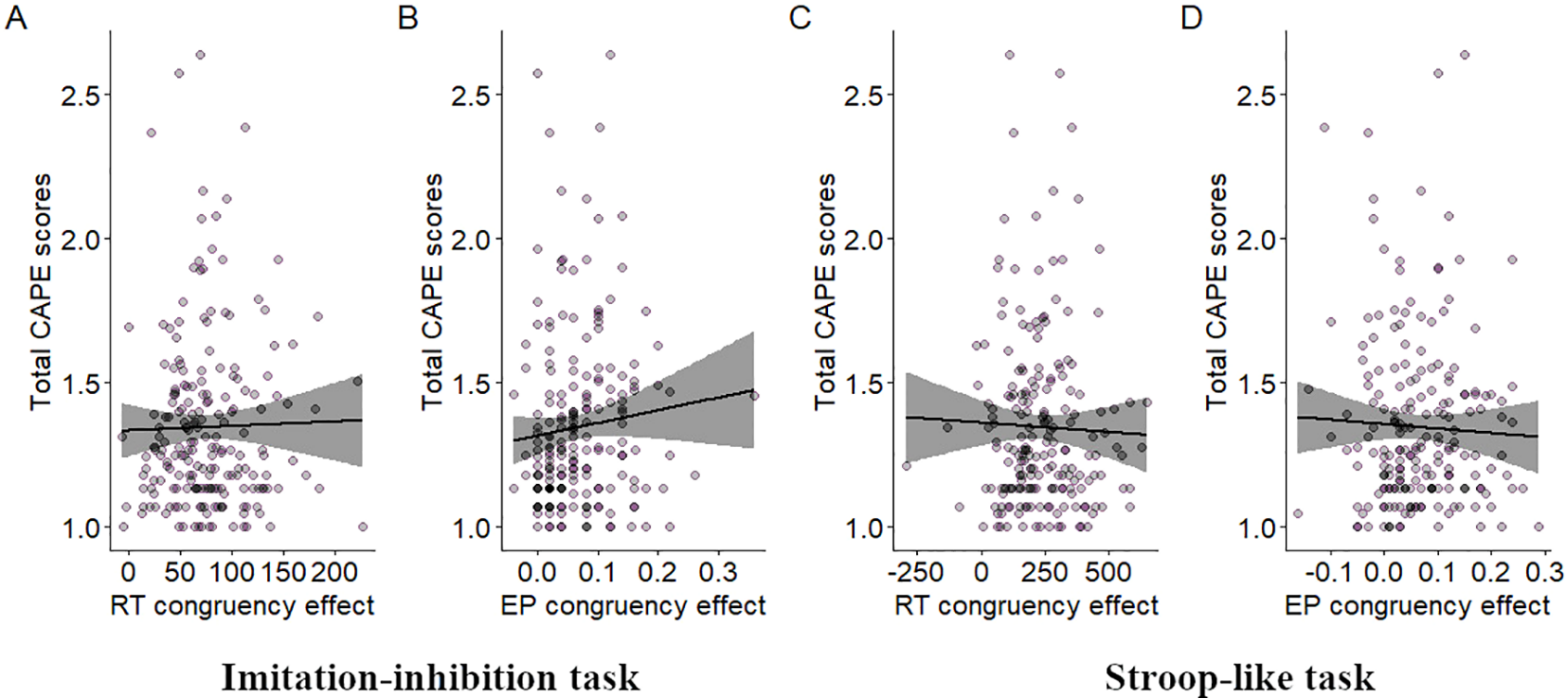
Relationship between total CAPE scores and inhibitory control. Dispersion plot between total CAPE scores and RT congruency effects in the imitation-inhibition **(A)** and the Stroop-like **(C)** tasks and between total CAPE scores and EP congruency effects in the imitation-inhibition **(B)** and the Stroop-like **(D)** tasks. A lower congruency effect reflects better inhibitory control. A lower value in the total CAPE scores reflect lower PE. Each point represents a single participant.

**Figure 3:**
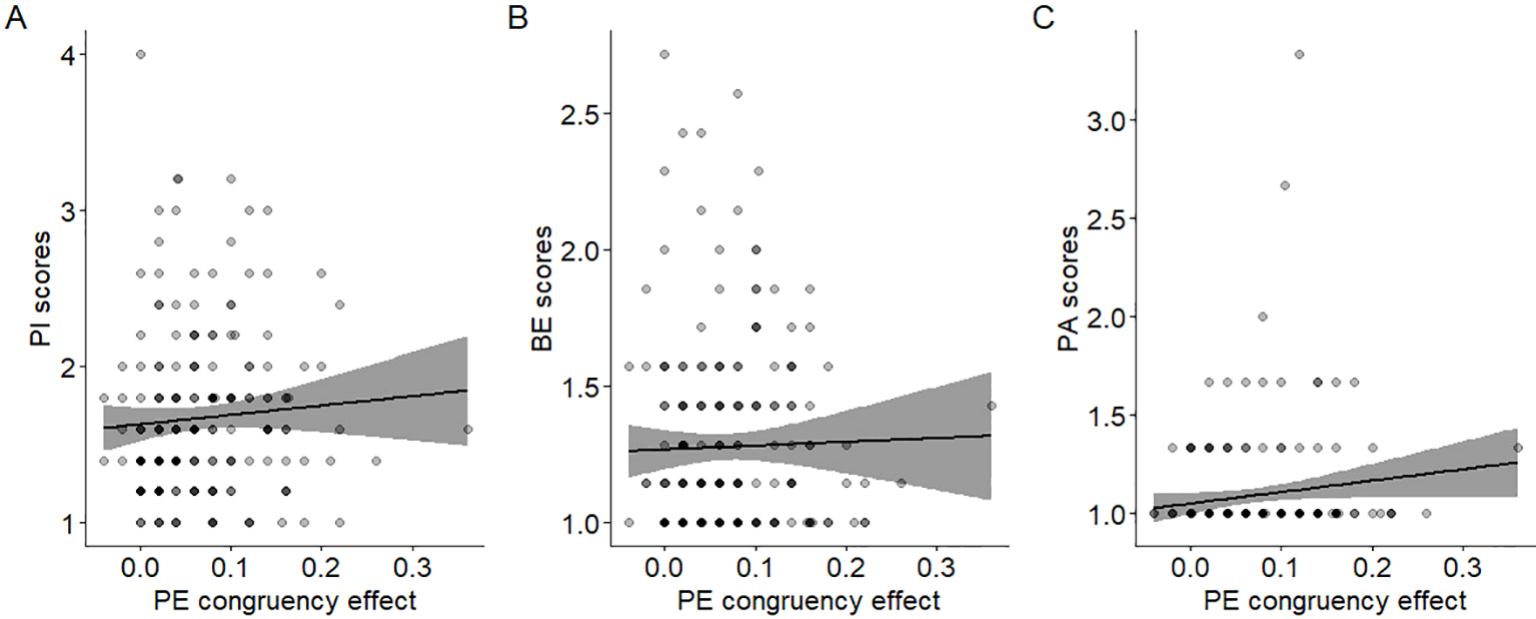
Relationship between CAPE subscale scores and inhibitory control. Dispersion plot between the EP congruency effects in the imitation-inhibition tasks and the **(A)** PI (r = 0.15 p = 0.027); **(B)** BE (r = 0.1 p = 0.14) and **(C)** PA (r = 0.09 p = 0.18) subscale score of the CAPE. A lower congruency effect reflects better inhibitory control. A lower value in the CAPE scores reflect lower PE in that specific dimension. Each point represents a single participant.

For the imitation-inhibition task no correlation was found between CAPE scores and congruency effects on RT (r = 0.007, p > 0.05; **Figure 2A**). However, a significant positive correlation was observed between CAPE scores and congruency effects on EP (r = 0.16, p = 0.025; **Figure 2B**). In the Stroop-like task no significant correlation was found between CAPE scores and congruency effects on RT (r = 0.02, p > 0.05; **Figure 2C**). Similarly, there was no significant correlation between CAPE scores and congruency effects on EP (r = 0.013, p > 0.05; **Figure 2D**). Overall, participants with higher CAPE scores demonstrated larger congruency effects on error proportions, implying increased difficulty in inhibitory control during incongruent trials. This relationship does not extend to the Stroop-like paradigm. We additionally found that the paranoid ideation scale was the only one that correlated with EP congruency effects (r = 0.15, p = 0.027; see **Figure 3A** and compare it with **Figure 3B** and **Figure 3C**).

## 4 Discussion

We examined associations between imitative inhibitory control and PE in individuals from the general population. We found that increased levels of PE are associated with greater error proportion-congruency effects. These findings suggest that alterations in the control of actions are associated with PE and align with prior evidence revealing motor abnormalities in schizophrenia patients (22) and healthy relatives of schizophrenic patients (23). This study contributes to the growing body of evidence supporting the idea that signs of psychopathology can be detected in healthy populations (16, 37). Importantly, our study suggests that imitative response control, rather than general inhibitory control, may be specifically related to PE.

Our study partially aligns with previous research linking psychotic experiences with deficit in inhibitory control in the general population, addressed by a Go/No-Go task (37). However, in our case, we observed specific changes associated with imitative behavior and no changes in general inhibitory mechanisms. These discrepancies might be attributed to differences in the age ranges examined. Since the gradual ontogenetic changes occurring in the prefrontal cortex (63, 64) and the subsequent maturation of inhibitory functions (65) in humans, it seems plausible that distinct inhibitory control mechanisms can be affected by psychopathology at distinct age ranges, complementing this previous study conducted at an early age state. Altogether, this evidence suggests we need to further explore inhibitory control as a potential mechanism in the development of psychopathology.

To succeed in the imitation-inhibition task participants must separate their inner motor representations from other’s motor representations. This has been taken as an index of self-other distinction (44). Since alterations of self-related processes are important in schizophrenia (66) using this task can give us clues of the mechanisms underlying psychopathology. However, previous attempts to understand schizophrenia using this task have been inconclusive. While Simonsen et al. (42) found enhanced imitation in schizophrenia patients, Rudolph et al. (43) observed an impaired control of imitation reflected in greater congruency effects. Although these results are in line with this latter finding, we found differences that were mainly concerned with accuracy rather than RT. While we anticipated observing changes in RT, we note that accuracy measures have proven to be more sensitive for diagnosing psychosis in some studies (67). In this sense, our findings emphasize that efforts should be made to report both RT and accuracy and also that we need to investigate the distinct contributions that accuracy and RT metrics offer to understand psychopathology.

Our key finding was that imitative inhibitory control is associated with PE. In order to dig into this effect, we exploratorily performed correlation analyses with the CAPE subscales and found that only paranoid ideation was statistically correlated with imitative inhibitory control (**Figure 3A**). This finding supports previous evidence suggesting that not all PE are equally relevant for psychopathology (68, 69). The fact that PI was the feature driving this association suggests that paranoia may be specifically related to some disturbances of imitative inhibitory control. This is in line with prior evidence revealing that psychomotor inhibition and paranoia show similar brain alterations (70). Since our task taps into a self-other distinction, its usage seems to be a promising approach to provide further insights on these associations.

We recognize limitations in our study. First, our cross-sectional design prevents us from making causal associations between variables. Second, self-report questionnaires addressing PE can bias participant’s answers, potentially inflating scores if normative experiences are also captured. However, CAPE prevalence reported here was similar to other studies investigating general population (71). Third, we used a convenience sampling method, which means that representativeness is not fully guaranteed. In this sense, our findings may not be generalizable to the entire population. However, it is important to note that this is an initial study aimed at exploring a possible relationship between imitative inhibitory control and psychotic experiences. While our sample may not represent the general population, it is a relevant subset for early-stage research, especially when studying phenomena that may be common in young, educated adults in Chile. Future studies will benefit from more diverse samples to assess whether the observed patterns hold across different sociodemographic groups. Fourth, the age-range of participants was relatively wide; however, we did not find correlations between PE and age. Fifth, we used an online version of the imitation-inhibition paradigm which could have potentially influenced the responses. Nevertheless, previous studies using this online version of the paradigm have found consistent congruency effects similar to those found in the live version of the paradigm (60, 72). Sixth, although the measurement scales are designed for general and subclinical populations, the variables may have presented some variability restriction in their lower range (i.e. floor effect), which could limit statistical power. This issue may be particularly relevant to the perceptual abnormalities dimension of the CAPE, which consists of only three items. Future studies should use a more comprehensive measure of perceptual abnormalities to reduce floor effects. Additionally, replicating these findings in a larger sample with more individuals reporting perceptual abnormalities would increase confidence in our current findings. Seven, although our effects are small, they are consistent with those reported in previous studies in the general population (e.g. 37). Finally, the exclusion criteria only screened out participants with a personal diagnosis of psychotic spectrum disorders, but did not assess schizotypal traits or relatives with psychosis. Schizotypal traits and family history of psychosis are known to be risk factors for PE, even in the absence of a clinical diagnosis (73, 74). We measured psychotic experiences using the CAPE, but this does not rule out the possibility that some participants may have had elevated schizotypal traits or a family history of psychosis. Future studies should include validated measures of schizotypal traits (e.g. the Schizotypal Personality Questionnaire) and assess more robustly family history of psychotic disorders.

In summary, we found that specific but not general inhibitory control abilities are associated with PE in a general population sample. This effect was exclusively evident for paranoid ideation. This preliminary evidence needs to be investigated more to disentangle the differential impact of distinct inhibitory control mechanisms in different age groups and populations. This could enhance our understanding of the transition to psychopathology and help us to anticipate clinical improvements in susceptible individuals.

## Data Availability

The raw data supporting the conclusions of this article will be made available by the authors, without undue reservation.
